# Expression of the Type VI Secretion System 1 Component Hcp1 Is Indirectly Repressed by OpaR in *Vibrio parahaemolyticus*

**DOI:** 10.1100/2012/982140

**Published:** 2012-07-31

**Authors:** Lizhi Ma, Yiquan Zhang, Xiaojuan Yan, Liping Guo, Li Wang, Jingfu Qiu, Ruifu Yang, Dongsheng Zhou

**Affiliations:** ^1^Department of Emergency Medicine, General Hospital of Chinese People's Armed Police Forces, Beijing 100039, China; ^2^State Key Laboratory of Pathogen and Biosecurity, Beijing Institute of Microbiology and Epidemiology, Beijing 100071, China; ^3^School of Public Health, Chongqing Medical University, Chongqing 400016, China

## Abstract

The type VI secretion system (T6SS) is bacterial protein injection machinery with roles in virulence, symbiosis, interbacterial interaction, antipathogenesis, and environmental stress responses. There are two T6SS loci, T6SS1 and T6SS2, in the two chromosomes of *Vibrio parahaemolyticus*, respectively. This work disclosed that the master quorum sensing (QS) regulator OpaR repressed the transcription of *hcp1* encoding the structural component Hcp1 of T6SS1 in *V. parahaemolyticus*, indicating that QS had a negative regulatory action on T6SS1. A single *σ* ^54^-dependent promoter was transcribed for *hcp1* in *V. parahaemolyticus*, and its activity was repressed by the OpaR regulator. Since the OpaR protein could not bind to the upstream region of *hcp1*, OpaR would repress the transcription of *hcp1* in an indirect manner.

## 1. Introduction

The type VI secretion system (T6SS) is recently discovered protein injection machinery found in at least one-fourth of all sequenced Gram-negative bacterial genomes [1–3]. Being used by pathogenic, symbiotic, and free-living bacteria, the T6SS is a versatile tool with roles in virulence, symbiosis, interbacterial interaction, antipathogenesis, and environmental stress responses. Conserved structural elements for the T6SS apparatus commonly include a transmembrane VipA/VipB tubular structure, a surface-exposed Hcp tube decorated with a VgrG trimer cap at the tip of the tube, and a ClpV homolog (a member of the AAA+ family of ATPases) energizing the system for protein secretion. The Hcp and VgrG proteins are transported out of the bacterium to assemble as a needle-likeinjectisomeon the bacterial surface, and the injectisome can puncture a vesicular membrane to translocate effectors into the host cell.

Quorum sensing (QS)  is a mechanism of gene regulation dependent on bacterial cell density [4–6]. Bacteria produce diffusible chemical signal molecules called autoinducers that accumulate when the cell density increases. When the stimulatory concentration of the autoinducers reaches a threshold, an alteration in the expression of many target genes occurs eventually via the acting of the master QS regulators. QS controls multiple cell functions, including symbiosis, virulence, competence, conjugation, antibiotic production, motility, sporulation, and biofilm formation.

*Vibrio parahaemolyticus* isone of the most important food-borne pathogens in coastal countries. *V. parahaemolyticus* harbors two T6SS loci, T6SS1 and T6SS2 [7]. The master QS regulator OpaR downregulates the T6SS1 genes, whereas it upregulates the T6SS2 genes as determined by a previous microarray expression analysis [8]. In the present work, the detection of the structural components Hcp1 for T6SS1 was achieved in a pandemic strain of *V. parahaemolyticus* O3:K6. The objective of this work was to characterize the OpaR-mediated repression of the *hpc1* transcription.

## 2. Materials and Methods

### 2.1. Bacterial Strains and Growth

The wild-type *V. parahaemolyticus* strain RIMD 2210633 (WT), kindly provided by Professor Mitsuaki Nishibuchi from Kyoto University, is a pandemic O3:K6 strain isolated from a patient with traveler's diarrhea in Japan in 1996 [7]. The entire coding region of *opaR* was deleted from RIMD 2210633 to generate the nonpolar *opaR* null mutant strain *ΔopaR* [9], using the suicide plasmid pDS132 by introducing homologous recombination [10]. 

For the common bacterial growth and maintenance, bacteria were cultivated in Luria-Bertani (LB) broth or on LB agar with addition of 2% NaCl at 37°C, and chloramphenicol was added at 5 *μ*g/mL when needed. For the longtime storage, bacteria were stored in Difco Marine (MR) broth 2216 (BD Bioscience) with addition of 30% glycerol at −85°C. For the bacterial growth of the following biochemical experiments, a two-round design of bacterial seed cultivation was employed: first, the glyceric stock of the bacteria was inoculated into 15 mL of the MR broth for growing for 12–14 h at 30°C with shaking at 200 rpm, and the cell culture was subsequently diluted to an OD_600_ value of about 1.0; second, the resulting culture was then diluted 50-fold into 15 mL of corresponding fresh MR broth and allowed to grow under the above conditions to reach an OD_600_ value of about 1.2–1.4. The bacterial seeds were then diluted 50-fold into 15 mL of corresponding fresh MR broth for further cultivation under the above conditions to reach an OD_600_ value of about 1.2–1.4.

### 2.2. RNA Isolation and Primer Extension Assay

For RNA isolation, the bacterial cell culture was mixed with two volumes of the RNAprotect (Qiagen) reagent. Total bacterial RNAs were extracted using the TRIzol Reagent (Invitrogen) [9, 11]. RNA quality was monitored by agarose gel electrophoresis, and RNA quantity was determined by spectrophotometry.

For the primer extension assay [9, 11], the oligonucleotide primer (5′-GAGTTTCACCGTTGATAGAC-3′) complementary to a portion of the RNA transcript of *hcp1* was employed to synthesize cDNAs from the RNA templates. About 10 *μ*g of the total RNA from each strain was annealed with 1 pmol of [*γ*-^32^P] end-labeled reverse primer using a Primer Extension System (Promega) according to the manufacturer's instructions. The same labeled primer was also used for sequencing with the fmol DNA Cycle Sequencing System (Promega). The primer extension products and sequencing materials were concentrated and analyzed in a 6% polyacrylamide/8 M urea gel. The result was detected by autoradiography (Kodak film).

### 2.3. Preparation of Purified OpaR Protein

Preparation of the purified OpaR protein was performed as previously described. The entire coding region of the *opaR* gene of strain RIMD 2210633 was directionally cloned between the *BamH*I and *Hind*III sites of plasmid pET28a (Novagen). The recombinant plasmid encoding the 6× His-tagged OpaR protein (His-OpaR) was transformed into *Escherichia coli *BL21*λ*DE3 cells. Expression of His-OpaR was induced by the addition of 1 mM IPTG (isopropyl-b-d-thiogalactoside). The overproduced protein was purified under native conditions using an Ni-NTA Agarose Column (Qiagen). The purified protein was concentrated with the Amicon Ultra-15 centrifugal filter device (Millipore), and the protein purity was verified by SDS-PAGE.

### 2.4. LacZ Fusion and *β*-Galactosidase Assay

A 544 bp promoter-proximal DNA region of *hcp1 *  was obtained by PCR with the ExTaq DNA polymerase (Takara) using RIMD 2210633 genomic DNA as the template (the sense primer: 5′-GCGCGTCGACGCTATCGGGTGTAGACGCTG-3′ and the antisense one: 5′-GCGCGAATTCGAGTTTCACCGTTGATAGAC-3′). PCR fragments were then directionally cloned into the *Sal*I and* Eco*RI sites of the low-copy-number plasmid pHRP309 [12] that harbors a chloramphenicol resistance gene and a promoterless* lacZ *reporter gene. Correct cloning was verified by DNA sequencing. An empty pHRP309 plasmid was also introduced into each strain tested as the negative control. The *V. parahaemolyticus* strains transformed with the recombinant plasmids and the empty pHRP309 plasmid were grown as above to measure the *β*-galactosidase activity in the cellular extracts [11] using the *β*-Galactosidase Enzyme Assay System (Promega) [13]. Assays were performed with at least three biological replicates.

### 2.5. Preparation of Polyclonal Antibody against His-OpaR

The 2.0–2.5 kg New Zealand white rabbits were immunized with the His-OpaR protein (100 *μ*g/rabbit) emulsified with the Freund's complete adjuvant through the subcutaneous route, followed by the subcutaneous boost immunization every two weeks for another three times with the mixture of His-OpaR/Freund's incomplete adjuvant. The specific antibody in the serum was monitored by indirect enzyme-linked immunosorbent assay (ELISA). The blood was collected by the carotid bleeding under callisection and the serum was separated for IgG purification by the conventional method of saturated ammonium sulfate. The purity of the antibody was verified by SDS-PAGE and its quantity was measured by UV spectrometry.

### 2.6. SDS-PAGE and Immunoblot Analysis

Bacterial cultures were centrifuged at 12000 ×g for 5 min, and the cell pellets were suspended in 20 mM Tris-HCl pH 8.0 buffer and used for SDS-PAGE and immunoblot analyses. The protein samples were separated by sodium dodecyl sulfate—13% polyacrylamide gel electrophoresis (SDS-PAGE). Western blot analyses were performed as described previously [14] using the anti-Hcp1 polyclonal antibody.

### 2.7. Electrophoretic Mobility Shift Assay (EMSA)

A 544 bp promoter-proximal region of *hcp1* was amplified by PCR with the sense primer 5′-GCTATCGGGTGTAGACGCTG-3′ and the antisense one 5′- GAGTTTCACCGTTGATAGAC -3′. For EMSA [9], the 5′ ends of DNA were labeled using [*γ*-^32^P] ATP and T4 polynucleotide kinase. DNA binding was performed in a 10 *μ*L reaction volume containing binding buffer [1 mM MgCl_2_, 0.5 mM EDTA, 0.5 mM DTT, 50 mM NaCl, 10 mM Tris-HCl (pH 7.5), and 0.05 mg/mL poly-(dI-dC)], labeled DNA (1000–2000 c.p.m/*μ*L), and increasing amounts of the His-OpaR protein. After incubation at room temperature for 30 min, the products were loaded onto a native 4% (w/v) polyacrylamide gel and electrophoresed in 0.5× TBE buffer for about 50 min at 220 V. Radioactive species were detected by autoradiography after exposure to Kodak film at −70°C.

### 2.8. DNase I Footprinting

For DNase I footprinting [9], A 544 bp promoter-proximal DNA region of *hcp1* with a single ^32^P-labeled end was amplified by PCR with either sense or antisense primer being end labeled (the sense primer 5′-GCTATCGGGTGTAGACGCTG-3′ and the antisense one 5′-GAGTTTCACCGTTGATAGAC-3′). The PCR products were purified using the QIAQuick columns (Qiagen). Increasing amounts of His-OpaR were incubated with the purified, labeled DNA fragment (2 to 5 pmol) for 30 min at room temperature, in a final 10 *μ*L reaction volume containing the binding buffer used in EMSA. Before DNA digestion, 10 *μ*L of Ca^2+^/Mg^2+^ solution (5 mM CaCl_2_ and 10 mM MgCl_2_) was added, followed by incubation for 1 min at room temperature. The optimized RQ1 RNase-Free DNase I (Promega) was then added to the reaction mixture, and the mixture was incubated at room temperature for 40 to 90 s. The reaction was quenched by adding 9 *μ*L of stop solution (200 mM NaCl, 30 mM EDTA, and 1% SDS), followed by incubation for 1 min at room temperature. The partially digested DNA samples were extracted with phenol/chloroform, precipitated with ethanol, and analyzed in 6% polyacrylamide/8 M urea gel. Protected regions were identified by comparison with the sequence ladders. For sequencing, we used the *fmol* DNA Cycle Sequencing System (Promega). The templates for sequencing were the same as the DNA fragments of DNase I footprinting assays. Radioactive species were detected as previously described.

## 3. Results

### 3.1. Two T6SS Systems in *V. parahaemolyticus*

**Figure 1:**
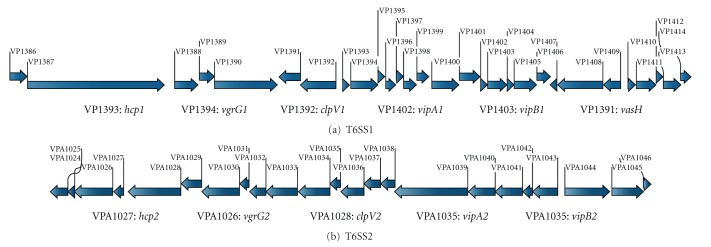
Organization of T6SS gene clusters in *V. parahaemolyticus*. The arrows indicated the present and gene order of the T6SS1 (a) and T6SS2 (b) loci on chromosomes I and II, respectively, which was based on the genome sequence of *V. parahaemolyticus* RIMD 2210633 [7].

Two large gene clusters (Figure 1) in the genome of *V. parahaemolyticus* RIMD 2210633 [7] potentially encode two different T6SS apparatuses (T6SS1 and T6SS2). The T6SS1 gene cluster (VP1386-1414) is composed of 29 consecutive genes forming 7 putative operons on the larger chromosome I, while the T6SS2 one (VPA1024-1046) consistis of 23 genes in 3 putative operons on the smaller chromosome II. Genes encoding the conserved structural elements Hcp, VgrG, ClpV, VipA, and VipB for the T6SS injectisome are found in both of the two gene sets, and they are further named as *hcp1 *  (VP1393), *vgrG1 *(VP1394), *clpV1 *(VP1392), *vipA1 *(VP1402), and *vipB1* (VP1403) for T6SS1 and as* hcp2 *(VPA1027), *vgrG2 *(VPA1026), *clpV2 *(VPA1028), *vipA2 *(VPA1034), and *vipB2 *(VPA1034) for T6SS2.

The *hcp1* is a 519 bp gene encoding a putative 172-amino-acid protein with an isoelectricpoint of 5.537, while *hcp2* is 480 bp in length encoding a 159-amino-acid with anisoelectricpoint of 4.702. The identity between the two Hcp alleles is only 15%. The *hcp1* gene was chosen for further analyses to explore the regulation of T6SS1 by the QS master regulator OpaR.

### 3.2. OpaR Represses Both Transcription and Expression of *hcp1*

**Figure 2:**
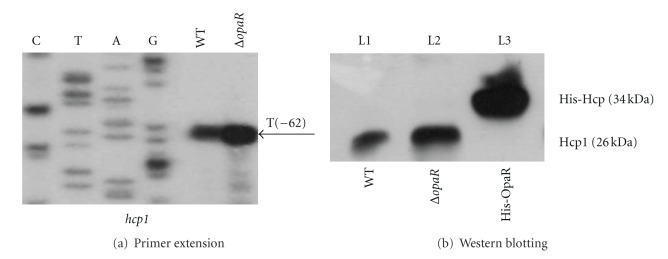
OpaR-dependent expression of Hcp1. (a) Primer extension. An oligonucleotide primer was designed to be complementary to the RNA transcript of *opaR*. The primer extension products were analyzed with 8 M urea—6% acrylamide sequencing gel. Lanes C, T, A, and G represent the Sanger sequencing reactions. The transcription start site of *hcp1* was underlined in the DNA sequences. (b) Immunoblot analysis.The Hcp1 production was detected in WT or *ΔopaR*, while His-Hcp1 was used as positive control.

The primer extension assay (Figure 2(a)) was conducted to detect the yield of the primer extension product of *hcp1* that represented the relative *hcp1* mRNA levels in WT and *ΔopaR*. A single-primer extension product was detected, and thus a single promoter was transcribed for *hcp1*, and its activity was under the negative control of the OpaR regulator. In order to further analyze how the expression of Hcp1 was regulated by OpaR, the whole cell lysate samples were taken for immunoblot analysis using anti-Hcp polyclonal antibody. As shown in Figure 2(b), the expression of Hcp was enhanced in *ΔopaR*. Our findings indicated that both transcription and expression of *hcp1 *  were negatively regulated by OpaR in *V. parahaemolyticus*. 

### 3.3. OpaR Greatly Represses Promoter Activity of *hcp1*

**Figure 3:**
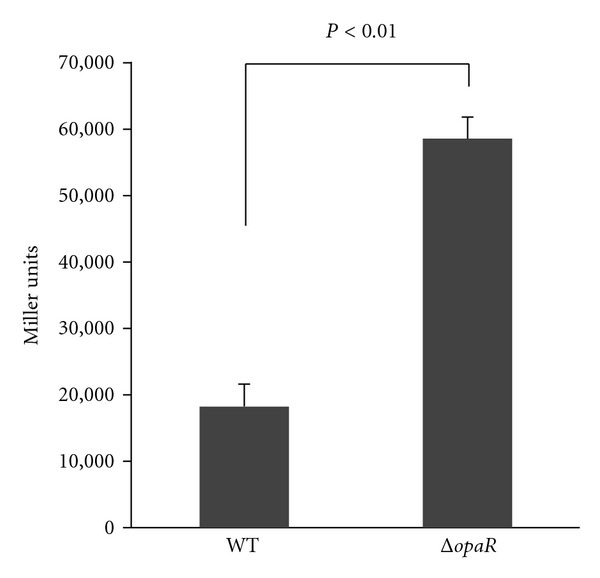
OpaR-dependent promoter activity of  *hcp1*. A promoter-proximal region 504 bp upstream to 40 bp downstream of *hcp1* was cloned into pRBR309 containing a promoterless* lacZ* reporter gene and then transformed into WT or *ΔopaR *  to determine the *β*-galactosidase activity in cellular extracts. Shown are the *hcp1* promoter activities (Miller units) in WT or *ΔopaR*.

To test the action of OpaR on the *hcp1 *  promoter activity, we constructed the transcriptional *hcp1::lacZ* fusion vector containing a 690 bp promoter-proximate region of *hcp1* and promoterless* lacZ* and then transformed into WT and *ΔopaR*, respectively. **β*-*Galactosidase activity was measured for evaluating the  *hcp1* promoter activity in WT or *ΔopaR* (Figure 3). The results indicated the **β**-galactosidase activity for the *hcp1::lacZ* fusion promoter in *ΔopaR* was much higher than that in WT. The empty LacZ reporter vector pHRP309 was also introduced into WT or *ΔopaR* as negative controls, and as expected, almost no **β*-*galactosidase activity was detected in each strain. These results indicated that OpaR repressed the *hcp1 *  gene at thetranscriptionallevel in *V. parahaemolyticus.*

### 3.4. OpaR Regulates *hcp1*  in an Indirect Manner

The 300 bp promoter region upstream of the start codon of *hcp1* was retrieved with the “*retrieve-seq*” program [15] and subsequently scanned with a previously constructed OpaR position frequency matrix (PFM) [9] with the “*matrices-paster*” tool [15]. The PFM, in which each row and column represents a position and a nucleotide, respectively, was thought to represent the conserved signals in the target promoter DNA regions for OpaR recognition [9]. This computational promoter analysis generated a weight score of 4.79 that was much lower than the generally used cutoff value of 7. This indicated that there was no cisacting OpaR consensus-like sequence within the *hcp1 *  promoter-proximal region.

**Figure 4:**
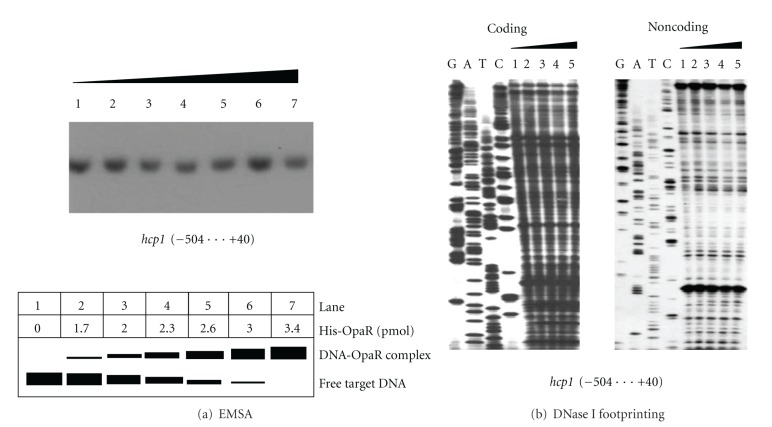
DNA binding analysis. (a) EMSA.The radioactively labeled DNA fragment from the 504 bp upstream to the 40 bp downstream of  *hcp1 *  was incubated with increasing amounts of purified His-OpaR protein (lanes 1, 2, 3, 4, 5, 6, and 7 containing 0, 1.7, 2.0, 2.3, 2.6, 3.0, and 3.4 pmol, resp.) and then subjected to 4% (w/v) polyacrylamide gel electrophoresis. Shown also was the schematic representation of the EMSA design. (b) DNase I footprinting. Labeled coding or noncoding DNA probes were incubated with increasing amounts of purified His-OpaR (lanes 1, 2, 3, and 4 containing 0, 6, 12.5, 18.8, and 25 pmol, resp.) and subjected to DNase I footprinting assay. Lanes G, A, T, and C represented the Sanger sequencing reactions. The footprint regions were indicated with vertical bars. The negative or positive numbers indicated the nucleotide positions upstream or downstream of  *hcp1*, respectively.

A 544 bp promoter-proximal region of *hcp1* was amplified, radioactively labeled, and subjected to EMSA with a purified His-OpaR protein (Figure 4(a)). The results showed that His-OpaR could not bind to this DNA fragment in a dose-dependent manner *in vitro*. DNase I footprinting experiments (Figure 4(b)) were subsequently performed with the 544 bp promoter-proximal region, which gave no footprint region protected by His-OpaR. In contrast, the His-OpaR protein recognized the upstream region of *opaR* (it was used as the positive control in the present work) in a dose-dependent pattern determined by both EMSA and DNase I footprinting (data not shown), which was identical to our previous results [9]. Taken all the above together, OpaR repressed the transcription of *hcp1* in an indirect manner, mostly likely through acting on another regulator that directly regulates the *hcp1 *  expression.

### 3.5. Structural Organization of *hcp1*  Promoter Region

**Figure 5:**
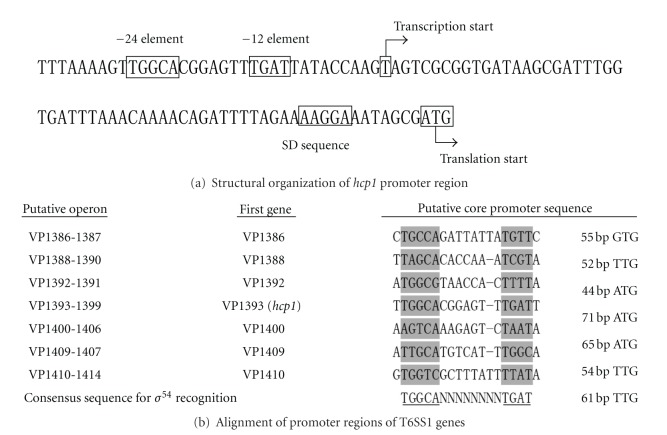
Organization of promoter-proximal regions. DNA sequence was derived from *V. parahaemolyticus* RIMD 2210633 [7]. Shown within the  *hcp1* upstream region (a) were translation and transcription starts, SD sequences, and −10/−12 and −35/−24 core promoter elements. The T6SS1 gene cluster forms 7 putative operons, and the upstream regions of the first genes of these operons were aligned for predicting the −10 and −24 elements (b).

The primer extension assay was able to map the 5′ terminus of the RNA transcript of *hcp1*, which allows one to determine the start site of transcription and helps to localize the core promoter region. The structural organization of the  *hcp1 *  promoter-proximal region was depicted (Figure 5), in which shown were translation and transcription starts, −35 and −10 core promoter elements for *σ* ^70^ (RpoD) recognition, −24 and −12 core promoter elements for *σ* ^54^ (RpoN) recognition, and Shine-Dalgarno (SD) sequence for ribosome recognition.

## 4. Discussion

The two complete T6SS loci in separate chromosomes of *V. parahaemolyticus* could encode two distinct T6SS apparatuses T6SS1 and T6SS2, since the putative counterpart conserved components (e.g., Hcp, VgrG, ClpV, VipA, and VipB) from the two T6SSs gave fairly low identity in amino acid sequences. The reciprocal regulation of T6SS1 and T6SS2 by the QS regulator OpaR [8] further indicated the two T6SSs would plays different cellular roles. As shown previously [16], a transposon insertion in  *hcp1* in the  *opaR* mutant background leaded to a severely impaired biofilm development phenotype. Both mRNA transcript (Figure 2(a)) and protein expression (Figure 2(b)) were detected herein for Hcp1 in the WT strain RIMD 2210633, indicating the expression and functionality of T6SS1 in pandemic *V. parahaemolyticus*. The functions of the two T6SSs in *V. parahaemolyticus* need to be further elucidated.

As shown in multiple bacteria [14, 17–23], QS appears to be a major regulatory mechanism for type VI secretion gene expression. The T6SS loci of *V. cholerae* (the causative agent of cholera) consist of the 17-gene *vas* cluster (VCA0107-0123), the *hcp1-vgrG1* operon (VC1415–VC1416), and the *hcp2-vgrG2* operon (VCA0017–0018) [24]. Mutation of the gene encoding the QS regulator LuxO leads to the enhanced expression and secretion of Hcp, which is dependent on the downstream regulator HapR that directly binds to the upstream promoter-proximal region of *hcp1* and *hcp2* to stimulate their expression [14, 25]. *V. alginolyticus* (an important fish pathogen) harbors two complete T6SS gene clusters (T6SSVA1 and T6SSVA2); the expression of Hcp1 in T6SSVA1 was positively and negatively regulated by the QS regulators LuxO and LuxR, respectively [17]. This work disclosed that the master QS regulator OpaR repressed the transcription of *hcp1*. 

The T6SS loci in various bacteria harbor the genes encoding the putative bacterial enhancer binding proteins (bEBPs; pfam family PF00158) [26], for example, the VasH regulator in *V. cholera* [27]. Both *σ* ^54^ and bEBPs are required for the expression of T6SS genes; the *σ* ^54^ binds to the consensus sequences centered at the −24 and −12 core promoter elements to recruit the RNA polymerase to the target promoters, while the cognate bEBP binds to the *cis*-acting upstream activating sequences of the target promoters [26]. The VasH homologue (VP1391) could be annotated in the genome of *V. parahaemolyticus* strain RIMD 2210633 [7]. In addition, the putative the −24 and −12 core promoter elements recognized by *σ* ^54^ could be found within the upstream regions of the first genes of all the seven putative operons of T6SS1 in *V. parahaemolyticus*. These indicated that the mechanism of *σ* ^54^ and VasH-dependent expression of T6SS1 was also employed by *V. parahaemolyticus*. 

The present work detected a single *σ* ^54^-dependent promoter transcribing for *hcp1 *  in *V. parahaemolyticus*. The activity of this promoter was repressed by the OpaR regulator, but the OpaR protein could not bind to the upstream region of *hcp1*; thus, OpaR repressed the *hcp1* transcription in an indirect manner. The computation promoter analysis with the OpaR PFM disclosed an OpaR consensus-like sequence within the upstream region of the VP1409–1407 operon (notably, VP1407 encodes a putative transcriptional regulator) rather than the *clpV1-vasH* (VP1392-1391) operon (data not shown). It was accordingly hypothesized that OpaR repressed the transcription of *hcp1* likely through acting on the VP1407 regulator (rather than VasH) that in turn regulated the *hcp1 *  expression.
